# Single-Step Plasma-Induced Synthesis of Graphene-Based Nanocomposites

**DOI:** 10.3390/nano16080473

**Published:** 2026-04-17

**Authors:** Neli Bundaleska, Edgar Felizardo, Ana Amaral Dias, Ana Maria Ferraria, Ana M. Botelho do Rego, Janez Zavašnik, Uros Cvelbar, Nenad Bundaleski, Pedro M. A. Guerreiro, Orlando M. N. D. Teodoro, Miroslav Abrashev, Jivko Kissovski, Amelia Almeida, Patrícia A. Carvalho, Thomas Strunskus, Bruno Gonçalves, Elena Tatarova

**Affiliations:** 1 Instituto de Plasmas e Fusão Nuclear, Instituto Superior Técnico, Universidade de Lisboa, 1049-001 Lisbon, Portugal; 2 Biospectroscopy and Interfaces Research Group, Institute for Bioengineering and Biosciences, Departamento de Engenharia Química, Instituto Superior Técnico, Universidade de Lisboa, 1049-001 Lisbon, Portugal; 3 Associate Laboratory i4HB—Institute for Health and Bioeconomy, Instituto Superior Técnico, Universidade de Lisboa, 1049-001 Lisbon, Portugal; 4 Department of Gaseous Electronics F6, Jožef Stefan Institute, 1000 Ljubljana, Slovenia; 5 Jožef Stefan International Postgraduate School, 1000 Ljubljana, Slovenia; 6 Centro de Física e Investigação Tecnológica (CEFITEC), Departamento de Física, Faculdade de Ciencias e Tecnologia, Universidade Nova de Lisboa, 2829-516 Lisbon, Portugal; 7 Faculty of Physics, Sofia University, 1164 Sofia, Bulgaria; 8 Centre of Physics and Engineering of Advanced Materials, Instituto Superior Técnico, Universidade de Lisboa, Av. Rovisco Pais 1, 1049-001 Lisbon, Portugal; 9 SINTEF Industri, Materials Physics, Forskningsveien 1, 0373 Oslo, Norway; 10 Chair for Multicomponent Materials, Institute for Materials Science, Christian Albrechts Universitaet zu Kiel, Kaiserstr. 2, 24143 Kiel, Germany

**Keywords:** graphene composite, N-graphene composite, microwave plasma, atmospheric pressure, scalable production

## Abstract

Graphene-based composite materials have attracted much attention for a range of applications in various fields, including electronics, sensing, catalysis, energy storage and conversion. Single-step large-scale microwave plasma synthesis of graphene and nitrogen-doped graphene (N-graphene) composite materials has been demonstrated. The developed atmospheric pressure plasma method allows continuous synthesis of different graphene-based hybrids in a controllable and environmentally friendly manner. Control over the synthesis process, i.e., size, uniformity, surface distribution of the nanoparticles and graphene/N-graphene quality, was provided by adjusting plasma parameters and injection configuration. Protocols for the production of particular composites, i.e., graphene-MnO, N-graphene-MnO, N-graphene-MnS, and N-graphene-Fe_x_O_y_, have been established using methane and acetonitrile as precursors. A comprehensive physicochemical characterization of the produced composites was conducted using high-resolution transmission electron microscopy, scanning transmission electron microscopy, Raman spectroscopy, X-ray diffraction, and near-edge X-ray-absorption fine-structure and X-ray photoelectron spectroscopies.

## 1. Introduction

Graphene-based nanocomposites, engineered through hybridization with nanoparticles, have drawn substantial research interest due to their distinctive catalytic, optical, mechanical, magnetic, and optoelectronic properties [1,2,3,4,5,6,7]. Frequently, the complementary advantages of the individual components lead to enhanced properties in the resulting composite, thereby significantly broadening its application scope. Different graphene-based nanocomposite combinations (with metal nanoparticles, polymers, inorganic nanostructures) have been developed and tested for applications in various fields, such as catalysis, electrochemistry, sensing, hydrogen storage materials, electromagnetic shielding materials, etc. [2].

The integration of graphene with various metal oxide particles in numerous configurations, such as anchored, wrapped, encapsulated, sandwiched, layered, and mixed architectures, resulted in a new class of advanced electrode materials. Graphene-metal oxide/sulfide hybrid structures, employed as electrode materials in energy storage and conversion devices, have consistently exhibited enhanced electrochemical performance relative to their individual constituents [2,8,9,10,11,12]. In addition to the synergistic effects present in graphene-metal oxide composites, the metal nanoparticles anchored on graphene sheets prevent agglomeration and restacking, thereby increasing the accessible surface area.

The outstanding physical and chemical properties of graphene-based composites have made them highly promising materials for photocatalytic pollutant removal. Coupling nanoparticles of titanium dioxide with a functional material like graphene is an effective method to enhance photocatalytic performance by extending its optical absorption range from the UV region to the visible region. Moreover, it was shown that the introduction of graphene can improve the charge-separation rate of TiO_2_. Photocatalytic degradation is a simple, effective, energy-saving and environmentally safe method to clean up pollutants [13,14,15].

Graphene-based composite thin films have also shown great potential in detecting key biological targets, including DNA, hydrogen peroxide, and glucose [16,17,18,19]. Additionally, metal nanoparticles (Mg, Li, Na, etc.) embedded within graphene matrices are being actively studied as hydrogen storage materials by employing their ability to form metal hydrides. Such materials enable reversible hydrogen adsorption and desorption processes, making them promising candidates for long-term hydrogen storage applications [20,21]. Moreover, graphene-based metal/metal oxide composites are investigated as potential antibacterial agents, electromagnetic radiation shielding materials, etc. [22,23,24,25]. In another example of synergy between Ti3C2Tx MXene nanoflakes and glycerol, enhanced lubrication performance was achieved, triggering liquid-superlubricity at the macroscale [26]. Additionally, novel DE@Mo_2_CTx MXene bio-microcapsule nanofluids demonstrated superior lubrication and heat dissipation [27].

Nitrogen-doped graphene (N-graphene) composites are particularly attractive due to their enhanced electronic structure and surface chemistry compared to pristine graphene composites. The introduction of nitrogen atoms into the graphene lattice generates additional active sites, improves electrical conductivity, and enhances surface wettability through the formation of nitrogen functional groups (e.g., pyridinic, pyrrolic, and graphitic N). These modifications facilitate stronger interfacial interactions, faster charge transfer, and better electrolyte accessibility. Consequently, nitrogen doping transforms graphene from a chemically inert conductor into a highly active, tunable material better suited for energy storage, catalysis, and electronic applications [28,29,30].

A large number of requirements exist for the quality of graphene and N-graphene-based composites in terms of structural integrity, purity, dispersion, functionalization, interfacial bonding and its integration into the host matrix. The quality of the materials plays a critical role in determining their performance across different applications and depends mainly on the fabrication techniques and the starting materials. The fabrication method itself must meet certain requirements to guarantee effectiveness, scalability, compatibility, and reproducibility. Additionally, manufacturing should ideally be a cost-effective and environmentally friendly process.

The strong interest in graphene-based composite materials, along with their wide range of potential applications, has driven extensive research and the development of new synthesis approaches [1,2]. Despite the continuous growth of the graphene and graphene-derivatives market, the industrial-scale production of these materials with high purity, well-defined properties, and cost-effectiveness remains a significant challenge. Recent findings highlight the substantial gap between laboratory-scale advances and commercial production [31]. Research efforts continue to face major challenges, particularly due to the lack of standardized and reliable protocols for producing high-quality, application-specific graphene and its derivatives on a large scale while maintaining consistent material performance.

To date, the synthesis of graphene/N-graphene-metal oxide/sulfide composites has been dominated by chemically driven approaches [2,32,33], including sol–gel [34,35], hydrothermal/solvothermal [36,37,38], self-assembly [39], and microwave irradiation [40,41]. Despite their widespread use, these methods often involve hazardous reagents that can adversely affect the structural and functional properties of the resulting nanomaterials. Solution mixing, for example, enhances the coating density of graphene with metal oxide nanoparticles (e.g., SnO_2_, TiO_2_); however, graphene oxide is commonly used as a precursor and the process promotes nanoparticle agglomeration, limiting uniform dispersion [42,43]. The sol–gel method enables in situ formation of uniform coatings with strong interfacial interactions and abundant nucleation sites; nevertheless, its typically multistep nature can lead to the formation of amorphous carbon and limited microporosity [34,35]. Hydrothermal/solvothermal techniques yield materials with high crystallinity and offer good scalability. However, the harsh reaction environments frequently introduce undesirable functional groups and reduced electrical conductivity [36,37,38]. Self-assembly approaches promote effective electrostatic interactions between the graphene and particles, yet they generally produce layered composites with predominantly amorphous characteristics [39]. Microwave irradiation provides a rapid ex situ deposition route, but it can result in the incorporation of unwanted functional groups and often necessitates additional post-synthesis purification steps [40,41].

Unlike conventional multistep chemical routes, our plasma-assisted approach enables the simultaneous synthesis of graphene, reduction of nanoparticle size, phase modification, and anchoring of nanoparticles within the graphene matrix, all achieved in a single step. This integrated process minimizes chemical waste and enhances process reproducibility. In this study, we present results from large-scale microwave plasma technology enabling controllable synthesis of graphene and N-graphene composites under atmospheric pressure conditions. The plasma-based process developed by our group, along with the corresponding prototype for synthesizing free-standing graphene-based structures, was presented in detail in [9,44,45,46,47,48]. In short, the process is based on gas-phase synthesis of free-standing 2D structures in the plasma environment, starting with decomposition of an organic precursor in the “hot” reactive plasma area into gas atoms and molecules and their further phase transformation into solid carbon in colder zones of the reactor. A narrow range of specific conditions (temperature gradients, precursors’ type and amount, reactor design, residence time) promotes the synthesis of the targeted planar graphene or N-graphene structures. While graphene/N-graphene are being synthesized in the upper part of the reactor, a controlled flow of nanoparticles (metal oxides or metal sulfides) is sprayed upstream into the reactive plasma medium. The nanoparticles are subjected to rapid heating and plasma-induced size reduction. Also, they participate in multiple chemical reactions with radicals, atoms, and molecules (H_2_, C, CN, HCN, etc.), which lead to the formation of different metal oxide phases and their anchoring to the growing graphene/N-graphene sheets.

This work presents the established protocols for the selective synthesis of graphene-based composites, such as graphene-MnO, N-doped graphene-MnO, N-doped graphene-MnS, and N-doped graphene-FexOy, using methane and acetonitrile as carbon and nitrogen precursors. The plasma prototype was tested under different operational parameters, including microwave power and precursor flows, as well as background gas under laminar and swirl flow injection regimes. The aim is to demonstrate the versatility of the plasma device, which provides a high level of customization via the application of special protocols, resulting in a controllable level of N-doping.

## 2. Materials and Methods

### 2.1. Plasma Prototype Machine (TRL-3)

**Figure 1:**
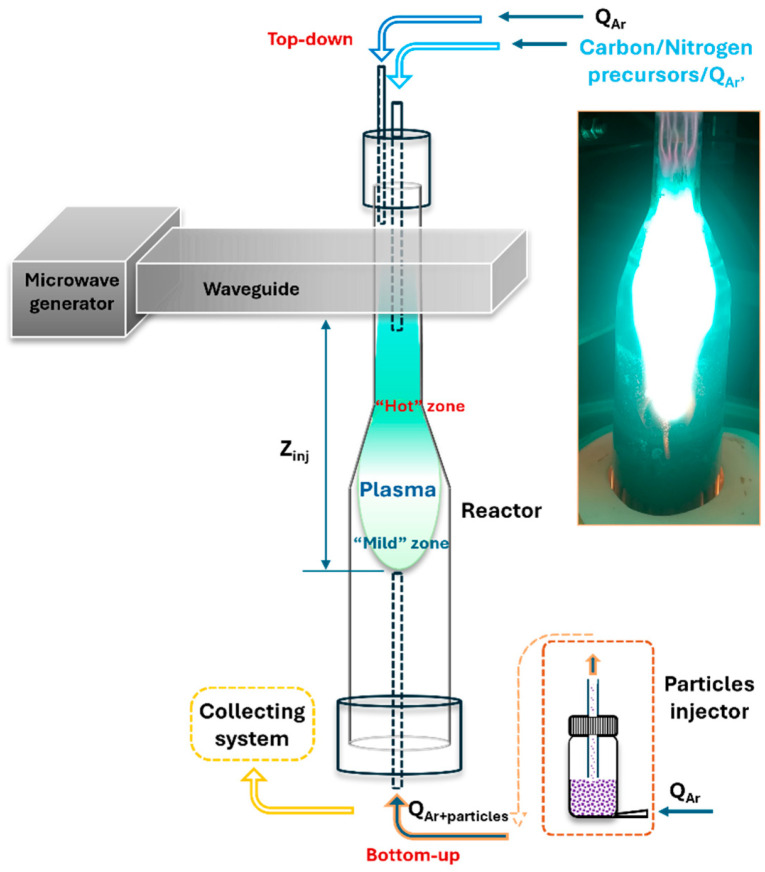
Schematic representation of the microwave plasma prototype used for synthesis of nanocomposites and photography of the plasma.

The custom-made plasma prototype machine, schematically illustrated in Figure 1, consists of four main components: a microwave generator and waveguide assembly, a quartz reactor tube with a gas injection system, a collection system for synthesized material, and a cooling system [44,48].

The electromagnetic waves at 2.45 GHz, emitted by the magnetron, travel through the waveguide and launch at the surfatron, a surface wave sustained plasma at atmospheric pressure conditions [49]. The surface wave propagates along the reactor at the interface between the reactor wall and the plasma. This configuration enables the plasma to extend beyond the launcher region, allowing for the injection of high microwave power densities and the generation of elevated concentrations of active species.

Background gas, argon, is injected near the top and along the inner wall of the reactor, while precursors are introduced through smaller-diameter quartz tube concentric with the reactor, providing their delivery in the “hot” zone of the plasma. Two injection modes are considered for the background argon gas, i.e., laminar and swirl. Another bottom-up line ending in a milder plasma zone (Z_inj_) delivers controlled flow of microparticles. This design enables fine control over thermodynamic parameters such as gas velocity, thermal flux, and residence time within the reactor.

The flow containing the synthesized free-standing nanostructures passes through a solid–gas separation system utilizing a tornado-based mechanism, which includes 2 to 5 collection containers for the accumulation of solid material. The plasma reactor is equipped with integrated water- and air-cooling systems to maintain continuous and stable operation of the entire prototype.

The process of nanostructure creation begins with the introduction of carbon/nitrogen containing precursor into the high-temperature (“hot”) plasma zone, sustained by the surface waves (Figure 1). Electrons absorb energy from the surface waves and transfer it to heavy particles through elastic and inelastic collisions, enabling gas temperatures of up to 3000 K. Under these conditions, the precursor molecules (methane) decompose into the fundamental building units of carbon nanostructures, namely carbon atoms and C_2_ radicals [46]. These species are then transported by the Ar flow into the mild afterglow zone, where the gas temperature decreases to approximately 500 K, and the gas-phase carbon atoms and C_2_ molecules transition into solid nucleation centers. This nucleation occurs within the larger volume of the mild zone, where kinetically driven processes govern the assembly and growth of free-standing, flowing carbon nanostructures [44]. To achieve N-graphene synthesis, acetonitrile was used as a precursor for both carbon and nitrogen. In the “hot” plasma zone due to the collisions, the acetonitrile decomposes through multiple chemical reactions (CH_3_CN → H + CH_2_CN, H + CH_3_CN → HCN + CH_3_, CH_3_ + CH_3_CN → CH_4_ + CH_2_CN), resulting in formation of active radicals, species, and atoms, such as H, CH_3_, HCN, etc. The HCN molecule is considered as the main “building block” of N-graphene. Graphene sheets are formed in the “mild” plasma zone [44].

The special reactor geometry with an expanding radius provides key advantages, such as injecting high-power density into its narrow part, necessary to process the large flux of precursors. Then, in the wider part of the tube, corresponding to the plasma mild zone, the residence time of the particles is prolonged, enabling their assembly into the desirable structures. In this region, the reduced density of carbon nuclei favors the formation of planar nanostructures. Finally, efficient assembly requires both rapid delivery and stacking of building units and sufficient energy input to overcome kinetic barriers, as well as precise control of the residence time of the levitating, growing nanostructures [44,45,46,47,48].

To produce nanocomposite materials, micron-sized particles (such as MnO_2_, oxy-MnS, or Fe_2_O_3_) are introduced into the reactor at a controlled rate. These particles are injected upstream at a specific location within the mild plasma zone, at the same time as graphene or N-graphene sheets are being formed. Once inside the plasma, the particles are rapidly heated to the surrounding gas temperature, which is approximately 2000 K. Under these conditions, the plasma acts as both a thermal and chemical reactor, enabling the micron-sized particles to undergo size reduction and transform into nanoparticles. In parallel, chemical interactions with reactive species (e.g., H_2_, O_2_, CN, HCN, etc.) lead to the formation of various chemical phases. The nanoparticles then attach to or become anchored onto the surfaces of the growing graphene or N-graphene sheets, forming hybrid nanostructures.

The introduction of swirl flow in the Ar background gas enhances mixing, stabilizes the plasma, and modifies the residence time and spatial distribution of reactive species. This directly affects the thermal distribution and local chemical environment experienced by the growing nanostructures, thereby influencing reduction processes, oxidation-state evolution of metal species, and nanoparticle anchoring on the graphene scaffold. Furthermore, the injection position of the precursors relative to the plasma zone governs the exposure of metal species to reactive radicals, which influences the phase transformations and the reduction of micron- to nano-size particles.

This process is highly efficient, which allowed us to produce tens of milligrams of free-standing graphene-based nanocomposites within just a few minutes. To ensure that the desirable properties of graphene are preserved, the synthesis conditions are carefully optimized. In particular, protocols are designed to maximize the 2D/G intensity ratio in Raman spectra, which is a key indicator of graphene quality and structural integrity. Moreover, the reproducibility is assessed by systematically analyzing each batch produced under selected operational conditions using XRD and Raman spectroscopy. The consistency of the resulting structural and phase characteristics across multiple batches confirms the reliability and reproducibility of the process.

### 2.2. Characterization Techniques

The morphology and crystal structure of the synthesized graphene-based nanocomposites were analyzed by Transmission Electron Microscope (TEM, JEM-2010F, JEOL Inc., Tokyo, Japan) operating at 200 kV. The visualization was performed in conventional and phase-contrast (HR-TEM) modes, and micrographs were recorded by a slow-scan CCD camera (ORIUS SC1000, Gatan Inc., Pleasanton, CA, USA). The chemical composition of the samples was assessed by Scanning Transmission Electron Microscope (STEM, Titan G2 60-300, FEI Inc., Eindhoven, The Netherlands) equipped with four energy-dispersive X-ray detectors (EDS, Super-X SDD) and high-angle annular dark field (HAADF) and bright field (BF) detectors. The samples were prepared by direct transfer of nanostructures onto commercial copper-supported lacy carbon grids without any further preparation.

Raman spectroscopy characterization was carried out on the synthesized nanostructures, which were freely suspended on a glass substrate. Spectra from various regions of the sample were acquired using a LabRAM HR Visible Raman spectrometer (Horiba Jobin-Yvon, Palaiseau, France) with a 633 nm excitation wavelength, a spectral resolution of 3 cm^−1^, and a laser spot size of 2 μm. To prevent sample overheating, measurements were performed at a low laser power of 0.054 mW.

The crystal structure and phase composition of the synthesized materials were analyzed using an X-ray diffractometer (XRD, D2 Phaser, Bruker Inc., Oeiras, Portugal) equipped with a CuKα radiation source (λ = 0.154184 nm). The powdered samples were manually pressed onto a zero-background sample holder to reduce background interference. Measurements were conducted at room temperature over a 2θ range of 5° to 95°. Data collection was carried out with a step size of 0.03° and an integration time of 5 s per step.

X-ray photoelectron spectroscopy (XPS) measurements of graphene-based nanocomposites were performed using a non-monochromatic dual-anode X-ray spectrometer (XSAM800, Kratos, Manchester, UK), employing Mg Kα radiation (1253.6 eV). Binding energies (BEs) were corrected for charge shifts by referencing the sp^2^ carbon peak at 284.4 eV. Quantitative analysis was conducted using the software library’s sensitivity factors. Additional operational parameters, acquisition settings, and data processing procedures are described in detail elsewhere [50]. XPS of N-doped graphene nanocomposites was performed on a home-assembled setup [51] that comprises an upgraded XPS setup (VSW Instruments, Manchester, UK) consisting of a non-monochromatic dual-anode X-ray source and the HA 100 hemispherical analyzer system. The peak fitting model for the C 1s line established in [52,53] was used, while the fitting parameters proposed in [54] were applied for the metal lines. The bond identification from the fittings of the O 1s and the N 1s line were performed in accordance with [53] and [55], respectively.

Near-edge X-ray-absorption fine-structure spectroscopy was performed on both graphene and N-graphene nanocomposites using the High Energy Spherical Grating Monochromator (HESGM) beam line combined with a ultrahigh vacuum chamber (PREVAC end station provided by Professor Ch. Wöll) at Helmholtz-Zentrum Berlin, Berlin, Germany (BESSY II storage ring). For free-standing graphene-based composites, no angular dependence of the NEXAFS spectra was observed. Spectra were recorded for the C, N, and O K-edges, as well as the Mn and Fe L-edges, using partial electron yield (PEY) mode with a home-built double-channel plate detector, probing a few nanometers in depth. The energy resolution E/ΔE used for the measurements was about 1000 leading to a 0.30 eV energy resolution at the carbon K-edge. The raw NEXAFS spectra were corrected for beamline transmission by normalizing against a reference spectrum of a clean, freshly sputtered Au sample. Energy calibration was achieved using an I_0_ feature at 284.9 eV measured at a carbon contaminated gold grid in the beam line which was aligned using the C 1s → π* resonance at 285.4 eV measured on a freshly cleaved graphite foil standard sample.

## 3. Results

### 3.1. Graphene-Based Nanocomposite

#### Graphene Manganese-Oxide Nanocomposite

Synthesis of graphene-metal oxide composite was achieved using methane as a carbon precursor, injected under laminar flow conditions in top-down configuration, and MnO_2_ microparticles (<10 μm). The following synthesis conditions were applied: Q_Ar_ = 9.4 Slm; Q_Ar’_ = 3 Slm; Q_CH4_ = 130 sccm; Q_Ar through MnO2_ = 200 sccm; Z_inj MnO2_ = 23 cm; and P = 4.86 kW, injecting manganese dioxide particles in the afterglow region using the bottom-up approach.

**Figure 2:**
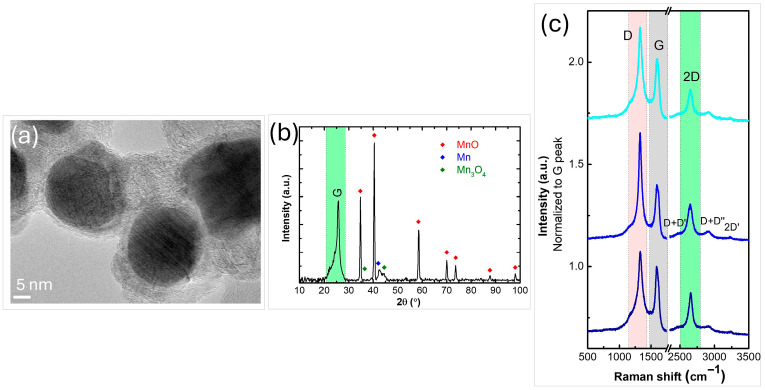
(**a**) HRTEM image; (**b**) XRD pattern; (**c**) Raman spectra of the synthesized graphene manganese-oxide nanocomposite.

The surface morphology and structural organization of the nanocomposites were studied by HRTEM analysis. A typical HRTEM image, illustrated in Figure 2a, indicates the presence of nanoparticles dispersed in the matrix of graphene sheets. The nanoparticles morphology and size distribution differ, with an average size in the range of 5–40 nm. The XRD pattern of the synthesized graphene-metal oxide composite displays multiple peaks, confirming the presence of various nanocrystalline phases. It shows the typical planes attributed to graphene sheets (labeled with G in Figure 2b) and to the cubic Fm3m MnO phase (PDF#07-0230, a = 0.4445 nm). Minor diffraction peaks marked with blue and green spots in the diffractogram are assigned to the Mn phase (PDF 21-0547) and tetragonal I41/and α-Mn_3_O_4_ phase (PDF #24-0734, a = 0.5762 nm), respectively.

The Raman spectra recorded from three randomly selected regions of the sample, as shown in Figure 2c, exhibit three prominent modes corresponding to the D, G, and 2D bands at approximately 1329, 1595, and 2650 cm^−1^, respectively. The presence of these peaks, especially the 2D peak, confirms the existence of graphene sheets in the sample. The G-band, originating from in-plane vibrations, appears in all sp^2^ carbon systems. The intensity ratio of the 2D to G peaks, along with the full width at half maximum (FWHM) of the 2D peak (FWHM~81 cm^−1^, I_2D_/I_G_~0.90), indicates the presence of multi-layered graphene sheets in the nanocomposite [56,57]. Although some inhomogeneity is expected due to nanoparticles being attached to and located between the graphene sheets, the Raman spectra collected from various random locations across the sample are nearly identical, indicating good overall homogeneity of the synthesized nanocomposite.

**Figure 3:**
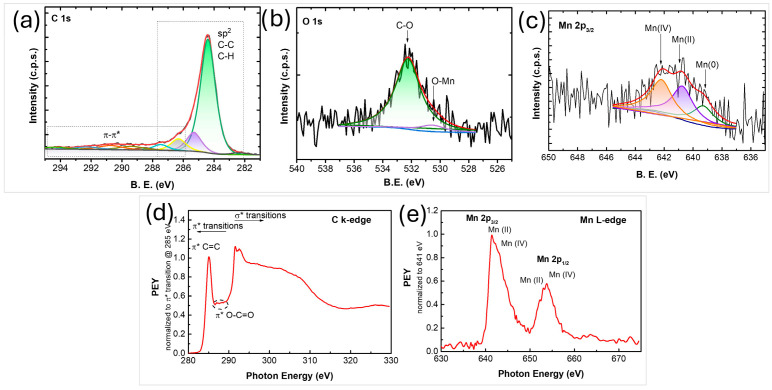
XPS spectra of (**a**) C 1s, (**b**) O 1s, (**c**) Mn 2p_3/2_ photoelectron lines; (**d**,**e**) NEXAFS spectra of the synthesized graphene manganese-oxide nanocomposite.

The XPS spectra of the synthesized nanocomposites reveals the presence of carbon, oxygen and manganese (Figure 3a–c). The results from the composition analysis showed 96.6 at% C, 3.1 at% O, and 0.3 at% Mn. The shape of the C 1s photoelectron line and its fitting contributions (Figure 3a) show two main features typical for an aromatic system, i.e., dominant peak centered at ~284.4 eV attributed to sp^2^ C-C and/or C-H and a wide hump in the range 287–295 eV corresponding to energy losses due to π-π* electronic excitations**.** Carbon atoms bonded to oxygen are included in the peak at ~286.4 eV. The peak in the O 1s region (Figure 3b) at ~530.4 eV is attributed to O-Mn bonds, while the one at ~532 eV is usually assigned to oxygen atoms bonded to carbon [53]. The peak observed at ~641 eV in the Mn 2p_3/2_ region (Figure 3c) corresponds to Mn and is shifted to lower binding energies compared to the corresponding peak of pure MnO_2_ introduced into the plasma (~642.1 eV) [9]. This shift suggests a partial reduction in MnO_2_ during the plasma process. Indeed, the formation of new phases, such as Mn(II) and Mn(0), was also confirmed by XRD analysis.

To further elucidate the chemical bonding environment within the synthesized nanostructures, NEXAFS spectroscopy was used, with both C K-edge and Mn L-edge spectra collected in partial electron yield (PEY) mode (Figure 3d,e). The C K-edge spectrum (Figure 3d) exhibits a distinct C 1s → π* transition at ~285.1 eV and σ* resonance near 292 eV, both characteristic of sp^2^-hybridized carbon. Additional features observed between 286 and 290 eV are typically attributed to structural defects or functional groups such as O–C=O, indicating the presence of oxygen-containing impurities within the graphene lattice [9]. The Mn L-edge NEXAFS spectrum exhibits two primary transitions corresponding to the 2p_3/2_ and 2p_1/2_ states, each of which can be fitted with two distinct contributions (Figure 3e). These are generally ascribed to Mn(II) and Mn(IV) oxidation states [58], consistent with the findings from XPS and XRD analyses.

Structural and spectroscopic analyses confirmed the formation of graphene sheets uniformly decorated with MnO, Mn, and Mn_3_O_4_ nanoparticles, with sizes ranging from 5 to 40 nm. Raman and XPS results demonstrated good structural homogeneity and the presence of few- to multi-layer graphene, while XRD and NEXAFS analyses revealed a mixture of manganese oxidation states, indicating partial reduction in MnO_2_ during plasma processing. The production rate of the graphene MnO composites is ~10 mg/min.

### 3.2. N-Graphene Based Nanocomposites

#### 3.2.1. N-Graphene Manganese-Oxide Nanocomposite

Acetonitrile, serving as a single-source precursor for both carbon and nitrogen, was injected in a top-down configuration directly into the hot plasma zone, enabling the synthesis of N-doped graphene sheets [44]. Concurrently, a precisely controlled jet of manganese dioxide (MnO_2_) microparticles was introduced into the upstream afterglow region to promote nanocomposite formation. The background argon gas was introduced through a swirl injection mode to enhance gas-phase mixing and stabilize the flow dynamics within the reactor. The following synthesis conditions were applied: Q_Ar_ = 7.4 Slm; Q_Ar through C2H3N_ = 150 sccm; Q_Ar through MnO2_ = 200 sccm; Z_inj MnO2_ = 23 cm; P = 5.4 kW.

**Figure 4:**
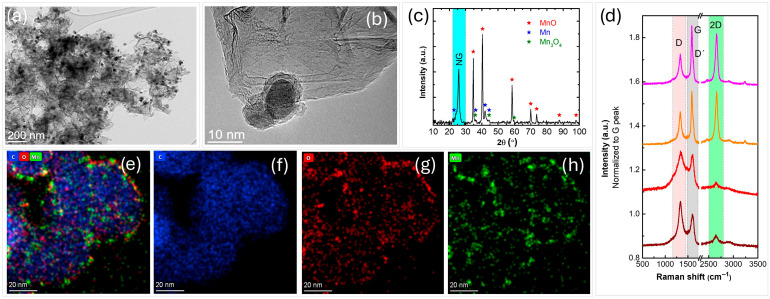
(**a**,**b**) HRTEM micrographs; (**c**) XRD pattern; (**d**) Raman spectra; (**e**–**h**) BF-STEM images and respective EDS maps of C (blue), O (red), Mn (green) of the synthesized N-graphene manganese-oxide nanocomposite.

The results from a comprehensive physico-chemical analysis of the manufactured nanocomposites are presented in Figure 4. HRTEM analysis, along with STEM imaging and corresponding EDS mapping of the N-graphene metal-oxide composites, reveal well-dispersed nanoparticles embedded within the N-graphene matrix. These nanoparticles exhibit a nearly spherical morphology with a size distribution predominantly below 100 nm (Figure 4a,b). The XRD pattern confirms the successful formation of nanocomposites (Figure 4c), with a high-intensity peak at 2θ~25.81° (002) designated as NG, indicating the graphene-based nature, newly formed phases including the dominant cubic Fm3m MnO (PDF#07-0230, *a* = 0.4445 nm), and minor diffraction peaks of Mn (PDF 21-0547) and tetragonal I41/and α-Mn_3_O_4_ phases (PDF #24-0734, *a* = 0.5762 nm). In comparison, injecting the nanoparticles closer to the hot plasma zone (Z_inj MnO2_ = 18 cm) favors synthesis of two phases, namely MnO (PDF 07-0230) and Mn (PDF 21-0547) [44]. Also, a lower intensity NG peak (002) was detected at the smaller precursor’s flow (Q_Ar through C2H3N_ = 90 sccm) [44], as compared to the current conditions (Q_Ar through C2H3N_ = 150 sccm).

The Raman spectra collected from four randomly selected spots of the nanocomposite sample are presented in Figure 4d. The characteristic D, G, and 2D bands typical of graphene-like materials are observed, although some regions exhibit a notably low-intensity 2D peak. This heterogeneity is expected due to the presence of nanoparticles attached to and intercalated between the graphene sheets, consistent with the HRTEM observations. The intensities of the D and D’ peaks, the latter appearing as a small shoulder on the G band, are directly related to the type and concentration of defects in the graphitic structure. The pronounced D band intensity therefore reflects both the incorporation of nanoparticles and nitrogen doping in the graphene lattice.

**Figure 5:**
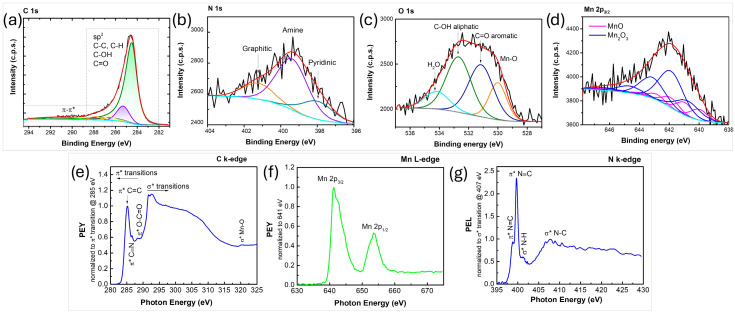
(**a**–**d**) XPS C 1s, N 1s, O 1s, and Mn2p_3/2_ regions, respectively; (**e**–**g**) NEXAFS C k-edge, Mn L-edge, and N k-edge spectra, respectively, of the synthesized N-graphene manganese-oxide nanocomposite.

High-resolution spectra of the C 1s, N 1s, O 1s and Mn 2p3/2 lines are presented in Figure 5a–d. As expected, the graphene scaffold is primary composed of carbon, nitrogen and oxygen, while manganese and parts of O partake in the manganese oxide phase. The C 1s line mainly consists of pure graphene (at 284.5 eV), contributing to about 83% of the total C 1s line intensity. Minor contributions from sp^3^-hybridized carbon or saturated hydrocarbons (C-C, C-H bonds) were also observed, representing about 11.5% of total intensity. C-N bonds are likely included within this contribution. The remaining carbon atoms are attributed to oxygen-containing functional groups, specifically C-O and C=O bonds. The N 1s line was fitted to three contributions, which can be attributed to pyridinic N, graphitic N and amine contributions. The O 1s line (Figure 5c) was fitted to four contributions. The narrow peak at approximately 530 eV is assigned to Mn-O bonds. The wider peaks at 531.2 eV and 532.7 eV were attributed to C=O bonds in the aromatic scaffold and aliphatic C-OH bonds, respectively. Finally, the contribution at 534.2 eV, accounting for 11.6% of the total O 1s intensity, is ascribed to adsorbed water. Compositional analysis of the N-graphene MnO nanocomposite indicated the presence of 90.4% of C, 6.5% of O, 2.2% of N, and 0.9% of Mn. When the two phases are considered separately, the graphene component consists of 93% carbon, 4.8% oxygen, and 2.3% nitrogen, while the MnO_x_ phase contains 56% oxygen and 44% manganese. These results are consistent with XPS analysis, which indicates that the MnO_x_ phase is composed of 43 mol% MnO and 57 mol% Mn_2_O_3_. It should be noted that XPS is a surface-sensitive technique, revealing only the composition of the surface region. These results do not necessarily represent the overall content of the MnO_x_ nanoparticles since their surface could be affected by the exposure to the discharge in the mild plasma zone.

NEXAFS analysis was used to probe the intra-molecular bonding of the produced nanocomposites (Figure 5). The C K-edge NEXAFS spectrum (Figure 5e) in PEY mode reveals the characteristic sharp C 1s → π* resonance at ~285.2 eV and σ* resonance at ~291.6 eV. Higher energy peaks result from transitions towards higher-lying states of π or σ symmetry. Features observed in the region of 286–290 eV are commonly associated with impurities within the graphene lattice, including doping. The residual peak observed at ~286.6 eV is attributed to π* C≡N and at ~288.1 eV to π* O–C=O. The peaks detected at ~320.1 and ~330 eV (not shown on the graph) are usually related to Mn-O presence [59]. Furthermore, Mn L-edge spectrum (Figure 5f) shows two distinct transitions, i.e., 2p_3/2_ and 2p_1/2_; these broadened peaks can be deconvoluted in three contributions that are frequently attributed to three oxidation states of manganese, namely Mn(II), Mn(III) and Mn(IV) [59,60]. The N K-edge spectrum (Figure 5g) displays several distinct peaks: those at 398.8 and 399.7 eV correspond to π* resonances associated with N=C and N≡C bonds, respectively, while the peaks at 401.4 and 407.9 eV are typically attributed to σ* resonances of N–H and N–C bonds, respectively [61].

N-graphene sheets decorated with well-dispersed manganese oxide nanoparticles formation were confirmed by comprehensive analyses. The plasma chemical reactivity resulted in modification of the metal microparticles into nano-sized ones and creation of new metallic phases (MnO, Mn, and Mn_3_O_4_). The observed defects and structural heterogeneity are attributed to both nitrogen doping and nanoparticle incorporation. The production rate of the N-graphene MnO composite at the conditions considered is ~15 mg/min.

#### 3.2.2. N-Graphene Manganese-Sulfide Nanocomposite

Acetonitrile, serving as a single-source precursor for both carbon and nitrogen, was injected into the hot plasma region to enable the assembly of N-doped graphene sheets. The background argon gas was introduced using a swirl injection mode to enhance mixing and flow stability. Subsequently, a precisely controlled jet of oxy-manganese-sulfide (MnS*) microparticles was sprayed into the plasma afterglow region to promote the formation of the nanocomposite. The nanocomposite was synthesized under following conditions: Q_Ar_ = 7.4 Slm; Q_Ar through C2H3N_ = 96 sccm; Q_Ar through MnS_ = 150 sccm; Z_inj MnO2_ = 23 cm; P = 5.6 kW.

**Figure 6:**
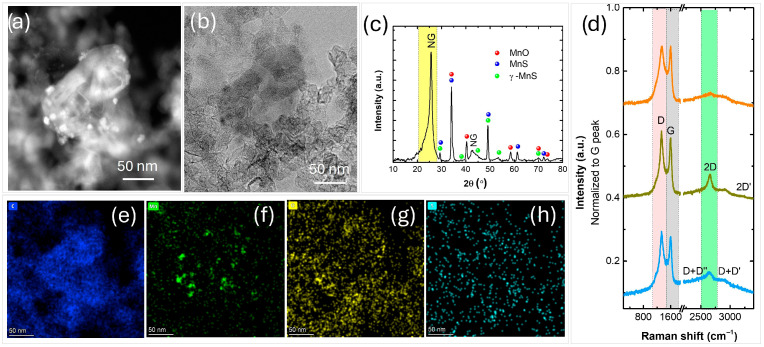
(**a**) HAADF; (**b**) BF-STEM image; (**c**) XRD pattern; (**d**) Raman spectra; (**e**–**h**) EDS map of C (blue), Mn (green), O (yellow) and S (turquoise) of the synthesized N-graphene manganese-sulfide nanocomposite.

The high-angle-annular dark-field (HAADF) STEM micrograph reveals graphene-like sheets decorated with nanoparticles of varying sizes, ranging from few to ~200 nm (Figure 6a). A bright-field BF-STEM micrograph along with the corresponding EDS elemental analysis (Figure 6b,e–h) confirms the presence of well-dispersed metal-sulfide nanoparticles on well-defined transparent graphene sheets within the synthesized structure. The X-ray diffraction pattern (Figure 6c) exhibits a prominent (002) diffraction peak at 2θ ≈ 25.62°, characteristic of N-doped graphene sheets. Based on the diffraction library data, three main crystalline phases were identified: MnS (PDF 40-1288), γ-MnS (PDF 40-1289), and MnO (PDF 07-0230). The presence of MnO is attributed to residual oxygen in the original MnS* precursor introduced into the plasma reactor.

Figure 6d presents Raman spectra acquired from three randomly selected locations on the sample. They exhibit the characteristic D, G and 2D bands associated with graphene-like materials except the first spectrum, which displays only a residual 2D peak. The pronounced D band intensity across all spectra suggests a high density of defects, likely arising from nitrogen incorporation into the graphene lattice and/or the anchoring of nanoparticles onto the graphene surface.

**Figure 7:**
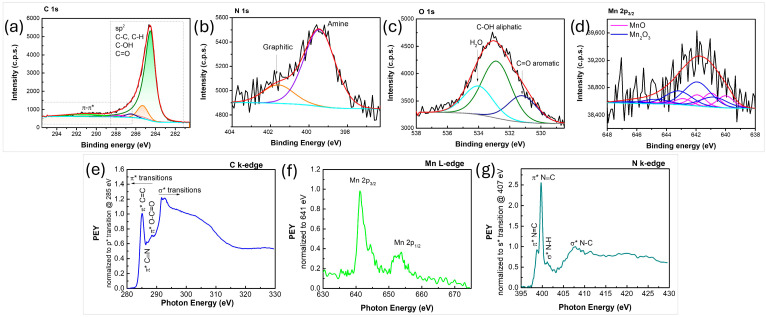
(**a**–**d**) XPS C 1s, N 1s, O 1s, Mn2p_3/2_ regions, respectively; (**e**–**g**) NEXAFS C k-edge, Mn L-edge, and N k-edge spectra, respectively, of the synthesized N-graphene manganese-sulfide nanocomposite.

XPS analyses reveal the presence of C, N, O and Mn, with their spectra presented in Figure 7a–d. The C 1s line is dominated by the graphene component at 284.5 eV, which accounts for approximately 87% of the total C 1s intensity. Minor contributions from sp^3^ hybridized carbon or saturated hydrocarbons (C–C, C–H bonds) are also observed (about 9.2%). Owing to the proximity of their binding energies, these components cannot be reliably distinguished. C–N bonds are also likely included within this contribution. The remaining carbon species are associated with oxygen-containing functional groups, namely C–O and C=O bonds. The N 1s line (Figure 7b) was fitted to two contributions, which can be attributed to graphitic N (19.4%) and amine (80.6%). The O 1s line was fitted with two broader contributions, centered at 531.2 eV and 532.8 eV, attributed to C=O groups within the aromatic framework and to aliphatic C–OH species, respectively, and a contribution at 534.0 eV, accounting for 25.5% of the total O 1s intensity, assigned to adsorbed water (Figure 7c). After prolonged acquisition using a twofold-higher pass energy (FAT 44 regime), Mn was eventually detected, whereas S remained undetectable (Figure 7d). This is not unexpected, given the significantly lower sensitivity of the XPS setup to sulfur compared to manganese (by more than a factor of three). Although Mn was detected, its chemical state could not be unambiguously determined, but the data clearly excludes the presence of metallic manganese. The spectrum can be tentatively fitted with a combination of MnO (53 mol%) and Mn_2_O_3_ (47 mol%). Overall, the results suggest that manganese is predominantly present in the Mn(II) oxidation state, which may correspond to MnS but could also arise from MnO. Furthermore, distinguishing oxygen associated with MnO_x_ species from that of the graphene scaffold in the frame of the O 1s line is not feasible due to the large disparity in their relative signal intensities. Compositional analysis of the N-graphene MnS composite indicated the presence of 93.8% of C, 4.5% of O, 1.6% of N, and 0.1% of Mn.

In contrast to the XRD and EDS results, where sulfur was detected, the XPS analysis did not reveal any detectable sulfur signal. Additionally, the relative manganese concentration was found to be very low. These observations are not contradictory, since XPS is a surface-sensitive technique probing only the top few nanometers of the sample. Additionally, it is also possible that the surface of MnS particles was modified during the discharge exposure within the mild plasma zone, building up an oxide interface.

NEXAFS measurements revealed the presence of carbon, nitrogen, oxygen and manganese in the sample. As expected, the C K-edge spectrum (Figure 7e) displays a π* resonance of aromatic C=C at ~285 eV. Additional features at ~286.6 eV and ~288.4 eV correspond to π* transitions of C≡N and O–C=O groups, respectively, confirming the presence of nitrogen- and oxygen-containing functional groups within the nanocomposite structure [61]. The Mn L-edge spectrum (Figure 7f) reveals the presence of Mn(II) and Mn(III) oxidation states, as determined through deconvolution of the Mn 2p peaks [9,53]. The N K-edge spectrum (Figure 7g) shows π* resonances of N=C (imine nitrogen) at ~398.8 eV, N≡C at ~399.7 eV, and σ* resonances of N–H at ~401.2 eV and N–C at ~407.9 eV.

The strategic introduction of MnS* microparticles into the plasma afterglow enabled the formation of well-dispersed metal-sulfide nanoparticles on N-graphene sheets. The incorporation of nitrogen and oxygen functional groups, along with mixed-valence manganese species, highlights the complex chemical environment within the nanocomposite, potentially enhancing its functional properties. The production rate of the N-graphene MnS composite at the conditions considered is 10 mg/min.

The produced by the present plasma technology nanocomposites were tested as electrodes for supercapacitors, i.e., an electrode based on the N-graphene metal-oxide(sulfide) nanostructures was developed with promising specific capacitances (~273 F·g^−1^ at 0.5 A·g^−1^) [9]. Analysis of the electrochemical charge storage properties of the developed nanocomposite materials demonstrated average specific capacitance values of 89.2 F·g^−1^ @ 0.5 A·g^−1^, a very good charge retention value of 76.3%, and excellent specific capacitance retention (88%) after 4000 cycles [9].

#### 3.2.3. N-Graphene Iron Oxide Nanocomposite

N-graphene nanocomposites were synthesized using acetonitrile as a carbon/nitrogen precursor and iron oxide (Fe_2_O_3_) microparticles injected up streams in the afterglow region of the plasma. The synthesis conditions include Q_Ar_ = 7.4 Slm; Q_Ar through C2H3N_ = 98 sccm; Q_Ar through Fe2O3_ = 150 sccm; Z_inj Fe2O3_ = 18 cm; P = 5.3 kW. The background Ar was injected under swirl flow arrangement, while acetonitrile was sprayed directly into the “hot” plasma zone.

**Figure 8:**
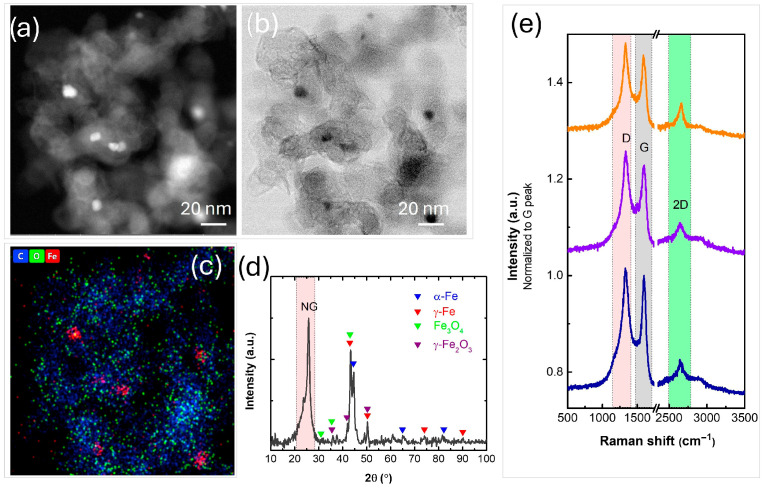
(**a**) HAADF and (**b**) BF-STEM micrographs; respective (**c**) EDS map of C (blue), O (green) and Fe (red); (**d**) XRD pattern; (**e**) Raman spectra of the synthesized N-graphene iron oxide nanocomposite.

HAADF and BF-STEM micrographs (Figure 8a–c) along with corresponding EDS elemental mapping reveal the presence of oxidized graphene sheets decorated with iron nanoparticles, predominantly below 10 nm in diameter. Under the applied plasma synthesis conditions, however, a uniform distribution of nanoparticles across the N-doped graphene framework was not achieved. The XRD pattern (Figure 8d) exhibits a prominent diffraction peak at 2θ~25.82°, characteristic of graphitic (002) planes. The dominant crystalline phases identified are α-Fe phase (PDF 06-0696) and γ-Fe (PDF 31-0619) with minor diffraction peaks identified as γ-Fe_2_O_3_ (PDF 25-1402) and Fe_3_O_4_ (PDF 19-0629). These findings are consistent with the morphological and compositional analyses provided by STEM and EDS. The Raman spectra collected from three random spots of the sample are nearly the same, indicating that a homogeneous sample of multi-layered graphene-based sheets (Figure 8e) was synthesized.

**Figure 9:**
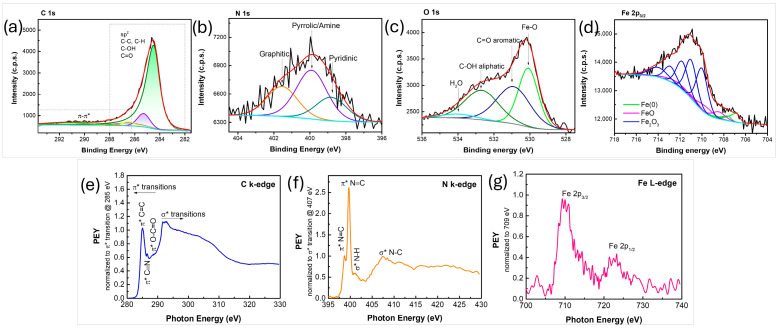
(**a**–**d**) XPS C 1s, N 1s, O 1s, and Fe 2p_3/2_ regions, respectively; (**e**–**g**) NEXAFS C k-edge, N k-edge, and Fe L-edge spectra, respectively, of the synthesized N-graphene iron oxide nanocomposite.

High-resolution spectra of the C 1s, N 1s, O 1s and Fe 2p_3/2_ lines are presented in Figure 9a–d. The graphene scaffold is composed primarily of carbon, nitrogen, and oxygen, whereas iron and a portion of the oxygen are associated with the iron oxide phase. The C 1s line mainly consists of pure graphene (typically at 284.4–284.5 eV), contributing to about 87% of the total C 1s line intensity (Figure 9a). Small quantities of sp^3^-hybridized carbon and/or saturated hydrocarbons (C–C, C–H bonds) were also detected (about 9.5%). We expect that C-N bonds are also part of this contribution. The remaining carbon atoms are bonded with oxygen in C-O and C=O bonds. The N 1s line was fitted to three contributions (Figure 9b), that can be attributed to pyridinic N (at 398.8 eV, 22.9%), graphitic N (at 401.5 eV, 29.4%) and amine contributions (at 399.9 eV, 47.7%) [55]. The fitting of the O 1s line enabled us to disentangle the metallic oxide contribution from the C-O bonds and the adsorbed water (Figure 9c). The narrow peak at approximately 530 eV corresponds to Fe-O bonds. The wider peaks at 530.9 eV and 532.7 eV were attributed to C=O bonds in the aromatic scaffold and aliphatic C-OH bonds, respectively [53]. Finally, a small contribution at 534.0 eV corresponds to adsorbed water. From the fitting of the Fe 2p_3/2_ line (Figure 9d), it appears that 8.6% of the detected iron atoms originate from the metallic iron, 14.3% originate from FeO phase and the remaining 77.1% from the Fe_2_O_3_ phase. Compositional analysis of the N-graphene FeO_x_ composite showed the presence of 89.8% of C, 6.4% of O (part of it being attributed to the FeO_x_ nanoparticles), 2.3% of N, and 1.5% of Fe. When the graphene phase is considered separately, it consists of 93.4% C, 4.2% O, and 2.4% N.

The NEXAFS measurements exhibited the presence of carbon, nitrogen, oxygen, and iron (Figure 9e–g), indicating successful incorporation of heteroatoms into the material. In the C K-edge NEXAFS spectrum (Figure 9e), a prominent π* resonance at ~285 eV is observed, corresponding to aromatic C=C bonds, while additional peaks at ~286.6 eV and ~288.3 eV are attributed to π* transitions of C≡N and O–C=O, respectively, reflecting the presence of nitrogen- and oxygen-containing functional groups. Furthermore, the Fe L-edge NEXAFS spectrum (Figure 9g) displays two distinct Fe 2p peaks, each of which can be fitted with two components corresponding to Fe(II) and Fe(III) oxidation states [62], which is in agreement with XPS results. The N K-edge spectrum (Figure 9f) further supports these findings, exhibiting π* resonances at ~398.7 eV and ~399.7 eV, corresponding to N=C and N≡C species, as well as σ* resonances at ~401.1 eV (N–H) and ~407.3 eV (N–C) [61].

Under the selected synthesis conditions, the synthesized nanocomposites demonstrated multi-layered, oxidized graphene sheets decorated with iron-based nanoparticles showing different phases (α-Fe, γ-Fe, γ-Fe_2_O_3_, and Fe_3_O_4_). While the nanoparticle distribution was non-uniform, Raman spectra confirmed overall structural homogeneity of the N-graphene scaffold. NEXAFS analyses provided additional validation: incorporation of nitrogen and oxygen functional groups, and identification of mixed oxidation states of iron (Fe^2+^/Fe^3+^). The production rate of the N-graphene FeO composite at the conditions considered was 15 mg/min.

## 4. Conclusions

The developed plasma method and the corresponding machine represent a disruptive, industrially relevant approach to synthesizing free-standing graphene, N-graphene, as well as related hybrids. This versatile, one-step, continuous process operates at atmospheric pressure and achieves gram-scale production without requiring post-processing or chemical treatments. Tailored protocols enable the selective synthesis of graphene and N-graphene nanocomposites using various precursors and ensuring reproducibility in production rate, structural quality, and chemical composition. The system efficiently produces hybrid nanomaterials by integrating metal oxide or metal sulfide nanoparticles into graphene/N-graphene matrices. Additionally, the reactive plasma environment enables micron-to-nano size reduction and phase conversion of metal oxide and sulfide particles. Controlled argon gas flow regimes allow fine-tuning of metal oxide nanoparticle size and composition.

Graphene-metal oxide hybrids were synthesized by injecting methane and micron-sized MnO_2_ particles into the mild plasma zone, while nitrogen-doped graphene composites were fabricated using acetonitrile as a dual carbon and nitrogen source.

A top-down injection of acetonitrile combined with bottom-up injection of MnO_2_ enabled the formation of N-graphene-metal oxide nanocomposites, featuring uniformly dispersed, nearly spherical nanoparticles (<100 nm) embedded within multilayered N-graphene sheets. Similarly, N-graphene-metal sulfide composites were produced via injection of MnS* particles, with plasma-induced reduction leading to the formation of distinct phases such as MnS, γ-MnS, and MnO.

Additionally, N-graphene-iron oxide nanocomposites were synthesized by controlled injection of Fe_2_O_3_ microparticles, resulting in α-Fe, γ-Fe, γ-Fe_2_O_3_, and Fe_3_O_4_ nanoparticles (~10 nm) embedded in a well-organized multi-layered N-graphene matrix.

The results demonstrate the versatility and efficiency of plasma-based methods for the direct synthesis of complex, free-standing graphene/metal oxide hybrid materials with tunable composition, morphology, and functional properties suitable for diverse applications. This plasma-assisted approach provides a strong foundation for sustainable, large-scale production of graphene-based hybrid nanostructures, offering precise control over material characteristics and broad potential for technological integration.

## 5. Patents

Process, (Reactor) and System production 2D nanostructures, INPI, PT 109387 B, 2021–12–14; Process, (Reactor) and System production 2D nanostructures, EPO, EP3455389, 2024–05–24; Process, (Reactor) and System production 2D nanostructures, USPTO, US 11,254,575 B2, 2022–02–22; Process, (Reactor) and System production 2D nanostructures, JPO, JP2019523695, 2022–08–01; Reactor production 2D nanostructures (divisional), INPI, PT 117332, 2022–05–12; Process, Reactor, System customization nanostructures, INPI, PT 110764 B, 2021–11–24; Process production nanocomposites, INPI, PT 115782 B, 2021–11–04.

## Data Availability

Data will be made available on request.
